# Possible Assessment of Calf Venous Pump Efficiency by Computational Fluid Dynamics Approach

**DOI:** 10.3389/fphys.2020.01003

**Published:** 2020-09-08

**Authors:** Gianni Niccolini, Andrea Manuello, Antonio Capone, Giuseppe Marongiu, Antonio Hector Dell’Osa, Andrea Fois, Fernanda Velluzzi, Alberto Concu

**Affiliations:** ^1^Department of Structural, Geotechnical and Building Engineering, Politecnico di Torino, Turin, Italy; ^2^Department of Mechanical and Aerospace Engineering, Politecnico di Torino, Turin, Italy; ^3^Orthopedic Clinic, Department of Surgical Sciences, University of Cagliari, Cagliari, Italy; ^4^Instituto de Desarrollo Economico e Innovación, Universidad Nacional de Tierra del Fuego, Antartida e Islas del Atlantico Sur, Ushuaia, Argentina; ^5^Biosignal Acquisition System, Nomadyca Ltd., Kampala, Uganda; ^6^Department of Medical Sciences and Public Health, University of Cagliari, Cagliari, Italy; ^7^2C Technologies Ltd., Academic Spin-Off, University of Cagliari, Cagliari, Italy

**Keywords:** calf venous pump, venous valvular incompetence, leg veins blood flow, computational fluid-dynamics, STAR CCM + platform

## Abstract

Three-dimensional simulations of peripheral, deep venous flow during muscular exercise in limbs of healthy subjects and in those with venous dysfunction were carried out by a computational fluid-dynamics (CFD) approach using the STAR CCM + platform. The aim was to assess the effects of valvular incompetence on the venous calf pump efficiency. The model idealizes the lower limb circulation by a single artery, a capillary bed represented by a porous region and a single vein. The focus is on a segment of the circuit which mimics a typical deep vein at the level of the calf muscle, such as the right posterior tibial vein. Valves are idealized as ball valves, and periodic muscle contractions are given by imposing time-dependent boundary conditions to the calf segment wall. Flow measurements were performed in two cross-sections downstream and upstream of the calf pump. Model results demonstrate a reduced venous return for incompetent valves during calf exercise. Two different degrees of valvular incompetence are considered, by restricting the motion of one or both valves. Model results showed that only the proximal valve is critical, with a 30% reduction of venous return during calf exercise in case of valvular incompetence: the net flow volume ejected by the calf in central direction was 0.14 mL per working cycle, against 0.2 mL for simulated healthy limbs. This finding appeared to be consistent with a 25% reduction of the calf ejection fraction, experimentally observed in chronic venous disease limbs compared with healthy limbs.

## Introduction

Proper flow of blood in the veins is important to ensure effective return of blood to the heart. However, the blood flow in the venous system is complex for several reasons: the low pressure values within the veins, flow rates varying from high values during muscular contraction to negligible flow during resting positions (Radegran and Saltin, 1998; Cassey and Hart, 2008), gravitational effects (Eiken et al., 2016), the collapsible nature of the venous wall (Kölegård et al., 2009), the presence of valves, and the large blood volume (about 64% of the total) that veins can hold due to their compliance.

Typically due to this, with prolonged relaxation of the muscle pump, the veins slowly fill via arterial inflow and become distended, allowing opening of the valves and eventually pressure increase (Stranden and Kroese, 1998) and it has been found that valvular dysfunction may occur in each venous system (superficial, perforating or deep) and in combination (Williams et al., 2014). For instance, venous dysfunction due to incompetent valves in both superficial and perforating veins causes a significant reflux in the superficial vein trunk and a reduction in superficial venous pressure smaller than normal, often referred to as “ambulatory venous hypertension” (Taylor and Steinman, 2010).

The purpose of this study was to contribute to identifying and validating a computational model that, using typical methods of the Computational Fluid Dynamics (CFD), allows the clinical experimenters, involved in solving vascular diseases, to optimize their diagnostic and therapeutic knowledge in patients with venous insufficiency of the lower limbs, caused by both primary pathologies of the venous structure (Eberhardt and Raffetto, 2014) and reduced or absent walking ability as a secondary effect (Crisafulli et al., 2009; Carrick-Ranson et al., 2013). Moreover, from that CFD model other medical and surgical specialties like, for instance, orthopedics and traumatology could obtain useful diagnostic and therapeutic information regarding the involvement of venous vessels in lower limbs suffering as a possible consequence of bones surgery or due to accidents with bone fractures of legs (Schmidt, 2017; Massari et al., 2018), while specialties such as metabolic syndrome and obesity could obtain more accurate and numerical information about the thrombosis risk in overweight patients (Stein et al., 2005; Borch et al., 2011).

## Materials and Methods

### Computational Fluid Dynamics Model

#### Geometrical Description

**FIGURE 1:**
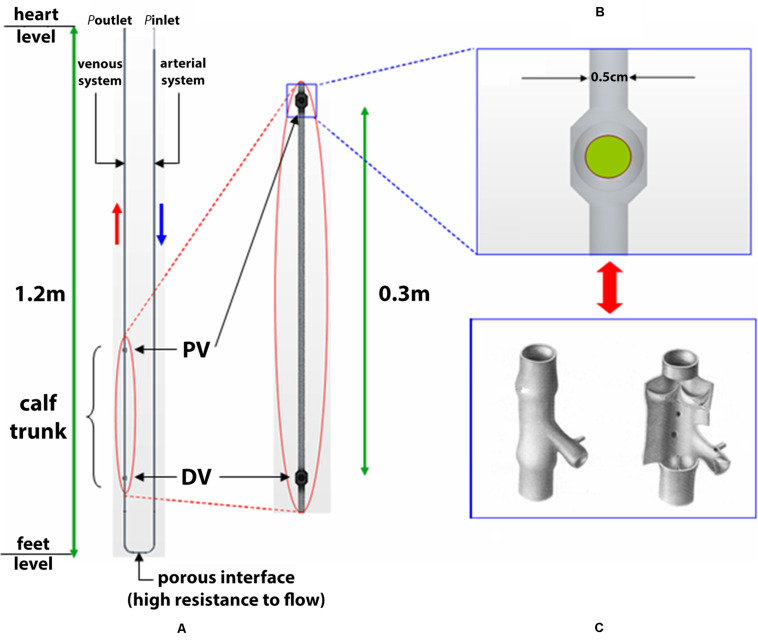
3D CAD schematic of systemic circuit. A simplified schematic is shown of the systemic circuit, conducting blood between heart and a lower limb given by the 3D computer aided drafting (CAD) model of a U-bend pipe with arms representing the venous and the arterial systems. The arms communicate via a porous baffle interface, which surrogates the resistance to flow offered by resistance vessels (small arteries and arterioles) yielding the physiological pressure drop from arteries through the capillary bed. Sketch represent the calf trunk with proximal valve (PV) and distal valve (DV) **(A)** and ball valve **(B)** simulating a venous valve **(C)**.

A simplified scheme of the systemic circuit is presented in Figure 1 where conducting blood between the heart and a lower limb, is given by the 3D computer aided drafting (CAD) model of a U-bend pipe with arms representing the venous and the arterial systems. The arms communicate via a porous baffle interface, which surrogates the resistance to flow offered by resistance vessels (small arteries and arterioles) yielding the physiological pressure drop from arteries through the capillary bed.

The circuit was sized so as to obtain blood velocities and pressures consistent with the physiological values in clinostatism reported in Table 1 (Berne and Levy, 1967).

**TABLE 1:** Average characteristic values for blood flow velocity and blood pressure in clinostatism.

**Vessel type**	**Velocity (cm/s)**	**Pressure (mmHg)**
Aorta	40–50	100
Arteries	10–40	95
Veins	0.3–5	15

The length of the circuit arms represents the distance heart - feet, set to 130 cm, to account for static pressures due to the fluid column in orthostatism. In particular, a variable pipe diameter was used to obtain the typical blood velocity for vessel typology, based on the continuity equation, *Av* = const (*v* is the blood velocity and *A* the vessel cross-sectional area).

For the sake of reproducibility, the diameters related to the vessel types of Table 2 are, respectively, 0.04 cm for the aorta (I), 0.08 cm for the arterial segment (II) and 0.5 cm for the calf vein (IV).

**TABLE 2:** Average blood velocity and pressure while simulating orthostatism for different circuit’s points (refer to Figure 4).

**Vessel type (and position)**	**Velocity (cm/s)**	**Pressure (mmHg)**
Aorta (I)	35	100
Arteries (II)	9.8	185
Veins (IV)	0.25	91

The current model, which reduces the parallel arrangement of blood circulation to a single vessel system, is mainly aimed at reproducing physiological values of blood velocity under different conditions (both static and dynamic). In this sense, the pipe diameters – calibrated using the continuity equation – are purely functional to the velocity calculation and do not correspond to physiological values.

#### Physics Description

The CAD model is then imported into the CFD software Star-CCM + (CD-adapco, New York, NY, United States) [https://mdx.plm.automation.siemens.com/star-ccm-plus], which uses the finite volume method. Two domains are considered in the model, specifically, the fluid domain in which the dynamics of the fluid are defined, and the solid domain in which the dynamics of spherical bodies, i.e., the ball valves, are defined. As regards the calf venous pump, periodic contractions and expansions of a deep vein (typically, the right posterior tibial vein) due to muscular work are simulated by a prescribed wall motion – via velocity boundary conditions – in a segment of the circuit. Hence, the vessel wall is rigid throughout the artery and vein, apart from in the region of the calf in the vein, where wall motion is prescribed. Here, the above-described pumping action is used to model a tip-toe movement repetition as being representative of a walking cycle. This trunk, considered with a length of 30 cm and a diameter of 0.50 cm, widens at its ends to form two ball valves which surrogate the function of venous valves and likewise certain mechanical models of the calf venous pump (Raju et al., 1993), as shown in Figures 1B,C. The closing member of each ball valve is a spherical ball with diameter, 0.52 cm, slightly larger than the vessel diameter, 0.50 cm. The use of ball valves is justified by the intention of studying the overall efficiency of the calf venous pump, and leaving out flow details near the valve. The characteristics of blood flow depend on several factors, including the vessels geometry, not only close to the area of interest, but also upstream and downstream of the vessel sections under examination. It may be necessary to take into account the reflection of pressure waves, the deformability of the blood vessel walls, and the branching of vessels as factors affecting the flow characteristics. Recently, it has been possible to numerically analyze complex models that take into account turbulent flows in transition often linked to cardiovascular disease (Behbahani et al., 2009).

On the other hand, simplifying assumptions in the blood flow model are allowed in a number of case studies, depending on the aim of the simulation. But, in any case, making simplifications requires a thorough knowledge of both the mechanics of fluids and the physiology of the cardiovascular system in order to quantify the deviations of model results from reality. There are research topics where assuming a Newtonian behavior for blood proved to be largely acceptable to the validity of CFD models results: this is the case study, for example, of ventricular assist devices (Givertz, 2011) or in the blood flow fields where the vessel diameter is bigger than a given value, involving a viscosity influence on the flow variables that is not as crucial as in the case of small diameters (Boron and Boulpaep, 2005). In large vessels such as the saphena, non-Newtonian effects are small and can generally be ignored. In general, blood behaves as a homogeneous Newtonian fluid in vessels with a diameter larger than one mm and shear rates ∂⁡*u*/∂⁡*y* over 100 s^–1^ (Truskey et al., 2004).

In this paper the blood is modeled as Newtonian incompressible fluid, with density ρ = 1060 kg m^–3^ and viscosity η = 0.0035 Pa-s, and laminar flow conditions are reasonably assumed (for blood vessels with radius *r* > 0.1 cm under non-pathological conditions). Axial symmetry can be assumed, as the effect of bend curvature on the flow are negligible in the segments of interest, including the horizontal one with the porous baffle interface and the vertical one with the valves. In this sense, the model is equivalent to a straight duct, embedded in a properly defined gravity vector field.

#### Initial and Boundary Conditions

Time-dependent boundary conditions are imposed on the circuit to mimic the cardiac and calf venous pumps. Pulsatile aortic pressure is imposed as a pressure inlet boundary condition, by assuming a sinusoidal law:

(1) $$P_{i\,n\,l\,e\,t}\,\left(t\right)=\left[100+20\,\sin\left(\frac{2\,\pi \,t}{T}\right)\right]\,m\,m\,H\,g$$

where diastolic and systolic values of 80 and 120 mmHg, respectively, and cardiac cycle *T* = 1 s are considered. A pressure outlet boundary condition *p*_*outlet*_ = 0 mmHg is imposed to simulate the pressure values close to zero in the vena cava. Instead, transverse contractions and expansions of the calf vein during walking are simulated by imposing a velocity boundary condition on the vessel wall, in the segment between the valves (see Figure 1). For this reason, the following radial velocity profile *v*_*r*_ (*z*,*t*) is used:

vr(z,t)={e⁢l⁢s⁢e⁢w⁢h⁢e⁢r⁢ez0≤z≤z0+L     0Vz⋅Vt⁢c⁢m⁢s-1

(2) $$v_{z}\equiv A\,\pi \,\sin\,\left[\pi \,\left(z-z_{0}\right)/L\right]$$

$$V_{t}\equiv T_{0}^{-1}\,\sin\,\left(\frac{2\,\pi \,t}{T_{0}}\right)$$

where *L* = 30 cm is the length of the deformable segment between *z*_0_ = 10 cm and *z*_0_ + *L* = 40 cm heights, *A* = 2.2⋅10^–5^ and *T*_0_ = 1 s is the period of wall motion. Initial values of velocity, *v*_0_ = 35 cms^–1^, and pressure, *p*_0_ = 100 mmHg [consistent with Eq.(1)], are chosen to initialize the iterative solver and, at once, to ensure rapid convergence toward physiological solutions (note the consistency between *v*_0_ and the aortic value of Tab. 2).

#### Meshing Model and Mesh Dynamics

With the governing Navier-Stokes for unsteady flows, the spatial fluid properties can be computed at each time step by implementing a finite element method, specifically an unsteady, implicit solver.

The time steps used to advance the solution are two: Δ*t*_1_ = 10^–2^ s to simulate slow dynamics in clino- and orthostatism, and Δ*t*_2_ = 2⋅10^–4^ s for faster fluid dynamics due to wall pumping mechanism.

The core of the fluid domain was filled by a polyhedral mesh with cell size of 5⋅10^–4^ m and, in the vicinity of wall boundaries, by three layers of prismatic cells (with cell thickness of 1.6⋅10^–4^ m) to model the boundary layer. The Courant-Friedrichs-Levy (CFL) condition for the stability assessment of numerical solution was applied. Recall that the CFL condition presently expresses that the distance covered by a fluid particle during the time step must be lower than the distance between mesh elements. If *v* is the velocity magnitude, Δ*t* is the timestep and Δ*x* is the length between mesh elements, i.e., the mesh size, the CFL condition takes the form: *C* = *v*Δ*t/*Δ*x* ≤ 1, where *C* is the Courant number. Taking Δ*t* = 2⋅10^–4^ s, *v* = 0.2 m/s, and Δ*x* = 5⋅10^–4^ m during calf pump activity, the CFL condition is largely fulfilled, being *C* = 0.08.

Then, the derived current pressure and viscous forces are used as input to calculate the balls’ motion.

Due to the movements of the boundaries — wall and balls— the mesh points in the fluid domain are displaced as well. Thus, before calculating pressure and velocity fields in the nodal points at the next time step, the mesh positions must be updated, by means of specific moving mesh techniques and remeshing procedures.

**FIGURE 2:**
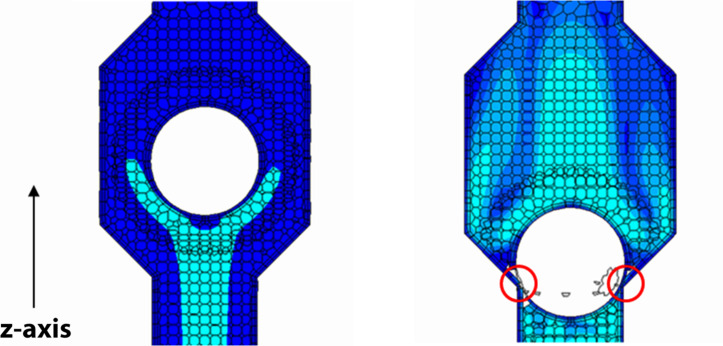
Ball valve with closing member in the two extreme positions under the action of the fluid. The overset mesh fitted around the ball is and overlapped to the background mesh; scalar representation of the blood velocity magnitude on a section plane of the valve (map color with lighter tones for higher velocity values). The closure defect (on the right marked by two circles) due to software limitations leaves open a ∼1% residual area of the area at full opening of the valve.

**FIGURE 3:**
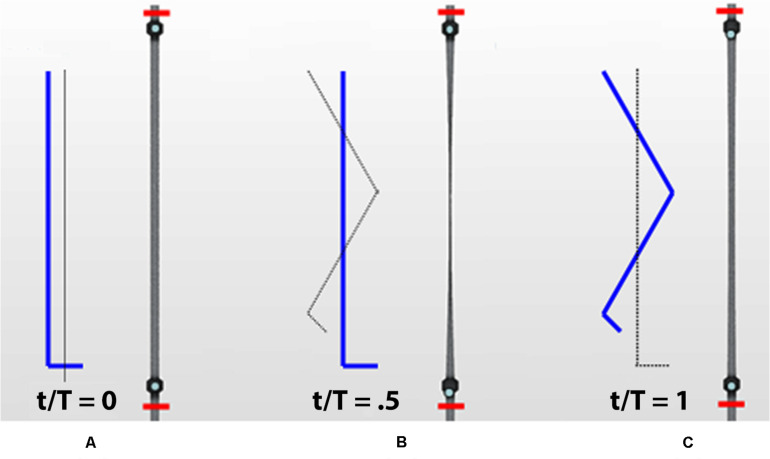
Different phases of the calf venous pump during a walking cycle. Contraction and subsequent relaxation of a calf vein in the leg marked in blue (red segments delimitate the studied calf trunk): initial orthostatism **(A)**, muscle contraction (systole) during weight-bearing **(B)** and muscle relaxation (diastole) during elevation of the leg **(C)**.

The interaction between rigid moving balls and fluid flow dynamics is solved by the overset mesh approach, where a moving a mesh fitted around the ball is overlapped to a stationary background mesh (Figure 2). Indeed, the venous segment at the calf level is made deformable using Eq. (2) as the imposed boundary condition for dynamic mesh morphing (shown in Figure 3). This procedure aims to mimic the behavior of flexible walls under the pressure of muscular contractions.

The mesh resolution is improved in the distal part of the housing valves, where the ball motion is prescribed. Because of limitations in the utilized software release (STAR CC + 8), balls must be stopped just short of the valve wall leaving a small gap (a 2-cell layer) for overset mesh to work correctly. Therefore, in order not to increase gap thickness in a manner which may cause valvular incompetence, the mesh is made finer. A mesh convergence study showed that model results are not affected by the mesh size for number of cells equal to or greater than 10^5^.

## Results

**FIGURE 4:**
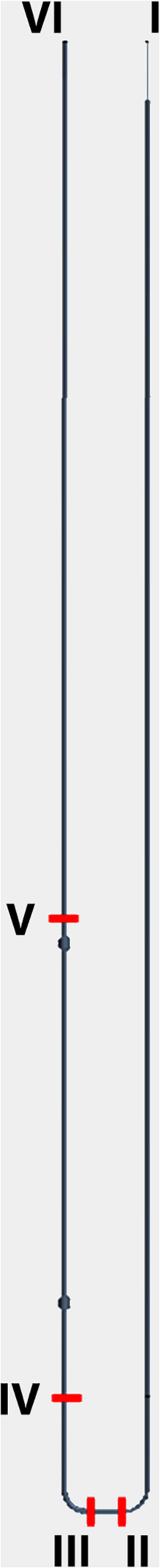
3D CAD. Model of systemic circuit with observation points (red dashes) of blood pressure and velocity. I: inlet of aortic trunk *P*_inlet_ = [100 + 20 sin (2πt)] mmHg; II: artery at feet level; III: vein at feet level; IV: upstream of the valvular system (ankle level); V: downstream of the valvular system; VI: outlet (vena cava) *P*_inlet_ = 0 mmHg.

The great variability in venous anatomy and function makes understanding of venous dysfunctions rather complex. Description of hemodynamics in lower extremity veins is greatly simplified here by the simple architecture of the circuit (Figure 4).

We reported the results of steady-state simulations, i.e., discarding the transient portion of output data affected by the initial conditions, concerning both healthy subjects and patients affected by different degrees of valvular dysfunction. The following diagrams depict pressure and flow volume vs. time in some relevant points along the circuit (Figure 4). Flow volumes are computed by averaging velocities across the respective vessel lumen.

Circuit sizing and boundary conditions properly chosen yield a good match between physiological and simulated pressure values under resting conditions. The observations at the beginning of all simulation runs, containing an initial transient phase, are discarded in order not to bias the outputs.

### Simulation 1: Clinostatism in Healthy Subjects

**FIGURE 5:**
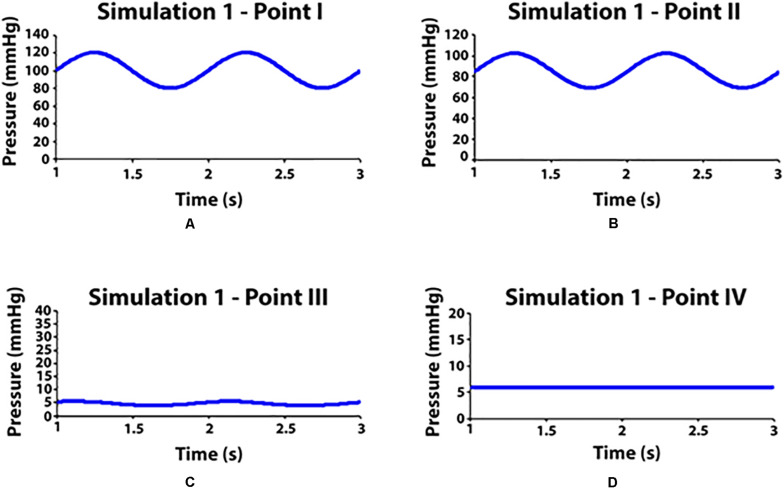
Pressure values measured in some observation points during simulation 1 (clinostatism). **(A–D)** Point I: aortic inlet; point II: artery at feet level; point III: vein at feet level; point VI: venous outlet.

Clinostatism is simulated by ignoring gravitational effects, i.e., de-activating the gravity model in the software physics. Hydrostatic contribution is negligible everywhere since all points are at heart level, and pressure variations are due to viscous flow (compare Figures 5A,B). Most pressure drop occurs at the level of the feet across the porous interface which separates arterial and venous sectors (compare Figures 5B,C). Note a residual modulation of venous flow (Figure 5C) at foot level, which is driven by the arterial pulsation in agreement with previous clinical studies (Joseph et al., 2016).

### Simulation 2: Orthostatism in Healthy Subjects

**FIGURE 6:**
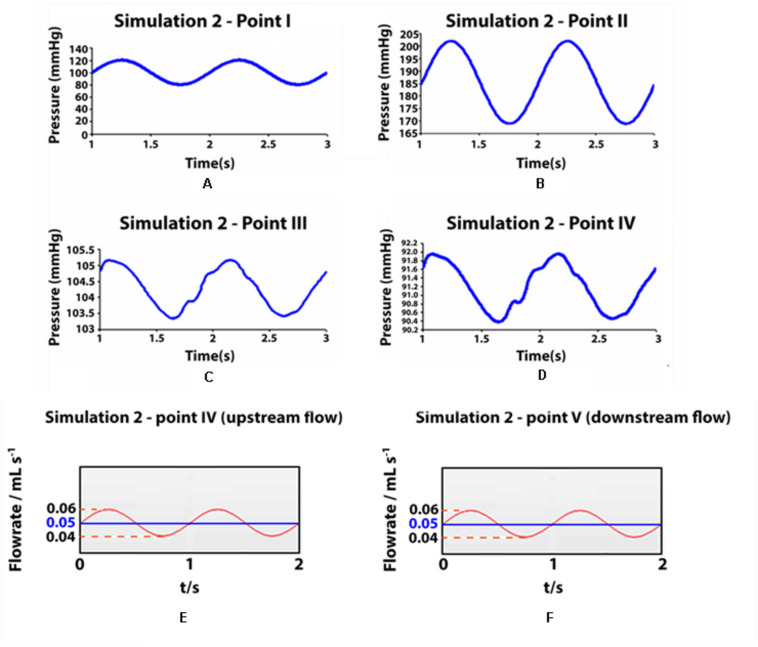
Pressure values and flow rates measured in some observation points during simulation 2 (ortostatism). **(A–D)** Pressure values measured in some observation points during simulation II (orthostatism). **(A–D)** Point I: aortic inlet; point II: artery at feet level; point III: vein at feet level; point IV: upstream of the distal valve (venous pressure at the ankle level). **(E,F)** Flow rate in two cross sections of the simulated deep vein, at ankle level **(E)** and at knee level **(F)** in orthostatism. The mean value represents also the integrated flow volume (0.05 mL) per cycle (T_0_ = 1 s) driven by one arterial pulsation.

In orthostatism, the hydrostatic pressure is greatly increased in the lower legs, giving an essential contribution to the total blood pressure (Figures 6B–D). In the venous sector of the circuit, valves remain open under the action of balanced forces on the balls. Thus, the venous pressure becomes approximately equal to (slightly higher than) the blood column pressure up to the heart level, as the kinetic contribution is negligible.

Flow measurements are performed in two cross-sections of the leg at the level of the knee and the ankle, i.e., downstream and upstream of the calf pump. Figures 6E,F shows a net volume of upward flow toward the heart, given by the integrated response to one arterial pulsation. The integrated flow volume per cycle (duration *T*_0_ = 1 s) is 0.05 mL through both cross-sections (Figures 6E,F), according to the mass conservation. The attenuation in the pulsatile regime of venous flow and pressure is apparent in Figure 6, where the values in the points III, IV and V range within very narrow intervals (highlighted by stretching the vertical axis).

### Simulation 3: Walking in Healthy Subjects

**FIGURE 7:**
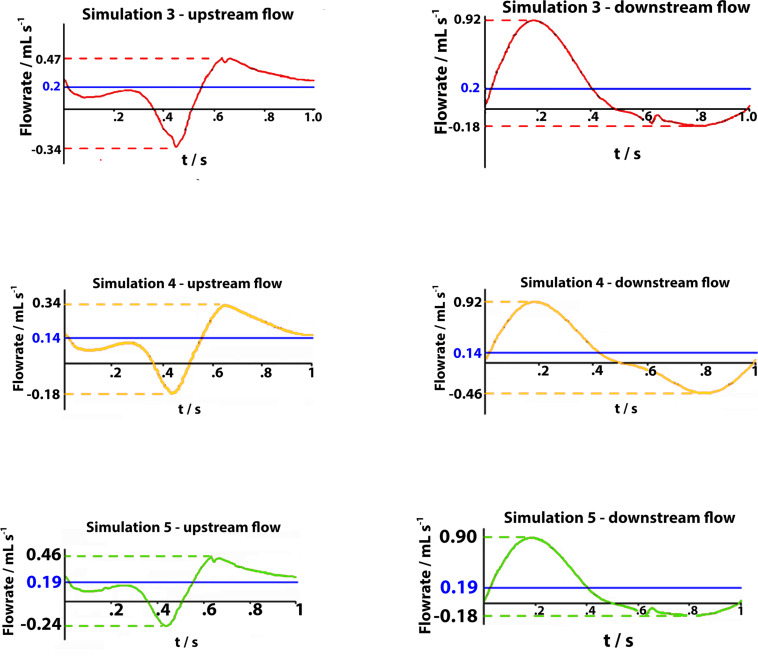
Graphs represent flow rates for 1 s walking cycle in healthy subjects (on the top), in subjects with severe dysfunction upstream of the distal valve and downstream of the proximal valve (on the middle), and in subjects with partial dysfunction upstream of the distal valve flow in downward direction (on the bottom). In each graph, time intervals: 0 < t < 0.5 s (on the left side), correspond to the muscle contraction (systole) during weight-bearing and the time intervals: 0.5 < t < 1 s (on the right side) correspond to the muscle relaxation (diastole) during elevation of the leg.

**FIGURE 8:**
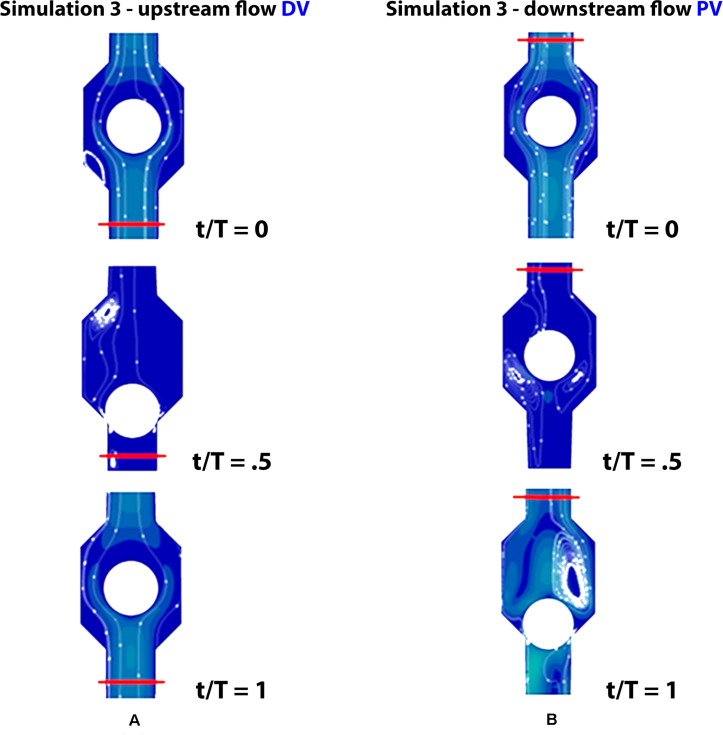
Valve positions during different phases of the walking cycle, lasting 1 s, in healthy subjects (simulation 3). Upstream of the distal valve i.e., at the ankle level **(A)** and downstream of the proximal valve, i.e., at the knee level **(B)**.

The observed net volume of upward flow toward the heart in response to the pumping action of the vessel is 4 times higher than in orthostatism (see graphs in Figure 7 on the top). The integrated flow volume per walking cycle (duration *T* = 1 s) is 0.2 mL, against the 0.05 mL value per one arterial pulsation during resting phases (note that these numerical values can be directly compared because T = T_0_). Flow measurements are performed in the two cross-sections downstream and upstream of the calf pump, whose mechanism is illustrated in Figures 3, 8A,B, whereas, unrealistic pressure diagrams are not reported. In fact, because of the absence of perforating veins in the simplified model, pressure at calf level greatly exceeds physiological values during closure of the valves, when dynamics is introduced.

Carrying out a mesh convergence study by varying the number of cells, it is found that at a point the response of the system converges to a solution, where refinement of the mesh has little to no effect on the solution. Taking the venous volume ejected from the calf per walking cycle as representative of the system response, a convergence study shows:

**Table:** 

Mesh characteristics (number of cells):	20000	50000	100000	300000
Results: integrated flow rate (mL/s):	0.3	0.22	0.2	0.2
Solve time (hh:mm):	00:05	00:07	00:09	00:19

### Simulation 4: Walking in Subjects With Severe Dysfunction

**FIGURE 9:**
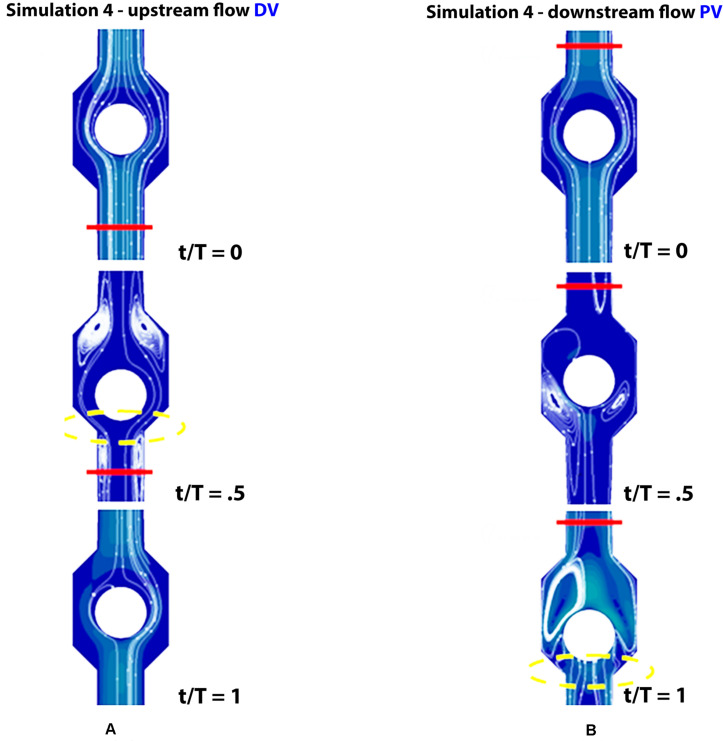
Valve positions in different phases of the walking cycle, lasting 1 s, in subjects with severe dysfunction (simulation 4). Upstream of the distal valve **(A)** and downstream of the proximal valve **(B)**, incompetent valves space are marked by yellow dashed lines.

Incompetent, or leaky, valves (implemented by restricting the ball motion, as shown in Figures 9A,B), affects the magnitude of retrograde venous flow. The flow rate in the steady state, i.e., in orthostatism, is clearly the same for healthy subjects (simulation 3) and patients with severe dysfunction (simulation 4). Different flow rates result only when the muscle pump is active and leaky valves can affect the venous return. In fact, this latter occurrence could significantly reduce the main stream from calf veins toward the inferior vena cava, thus influencing in a not negligible way the blood diastolic return to the right atrium. The incompetence degree of a leaky valve may be measured by the ratio of the sphere radius to the distance between the sphere in the lower position (see Figures 9A,B) and the vessel wall. Leakage is present also for competent valves due to the current software limitations, ranging from 0.16 mm for healthy subjects to about 2 mm in case of severe dysfunction. A parametric study would be necessary to express a precise correlation between reduction in venous return and this blockage ratio. A significantly increased reflux is observed downstream of the proximal valve (PV) during muscle relaxation (compare the magnitude of negative flow rates in diagrams of Figure 7 on the right top and middle), whereas, no changes are observed during muscle contraction (positive values in diagrams of Figure 7 on the right top and middle). That results in augmented bidirectional flow and reduction in ejected venous volume toward central direction, i.e., 0.14 mL per cycle, against 0.2 mL in healthy subjects.

### Simulation 5: Walking in Subjects With Partial Dysfunction

**FIGURE 10:**
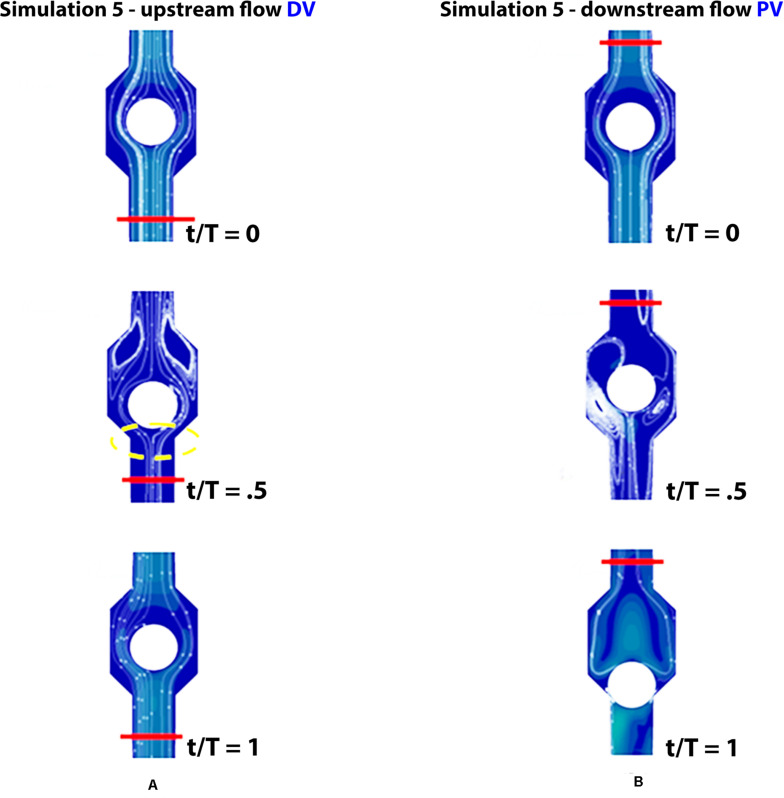
Valve positions during different phases of the walking cycle, lasting 1 s, in subjects with partial dysfunction (simulation 5). Upstream of the distal valve **(A)** and downstream of the proximal valve **(B)**, incompetent space of the upstream valve is marked by yellow dashed lines.

Incompetence of the distal valve is here combined with normal functionality of the proximal valve (Figures 10A,B). Such degree of dysfunction does not affect the venous return, with net flow volume in an upward direction of 0.19 mL per cycle (graphs in Figure 7 on the bottom), substantially identical to that of the physiological case.

## Discussion

Only within the legs does the venous system show its peculiar characteristics of having to move blood against the force of gravity. In the upright position, muscular contractions in the lower extremity squeeze venous blood in a central (proximal) direction, and the veins are refilled during the muscle relaxation phase (Keijsers et al., 2015). The presence of venous valves (bicuspid flap like structures) makes it possible to maintain the central direction of venous flow, in the event of zero or even negative pressure, and thereby to prevent downward flow. In this way, cyclic muscular action and one-way valves form a powerful pumping system aiding the venous return to the heart. The muscle pump exploits the great flexibility of the veins which collapse under external forces and distend under internal pressures. In fact, when the muscle tone of the legs is absent, as is the case for patients with spinal cord injuries, the diastolic return of the blood to the heart is strongly compromised and this can cause a reduction in cardiac output (Crisafulli et al., 2009) with a possible decrease in their aerobic work capacity.

From the energetic viewpoint, the kinetic term (which is proportional to the square of the velocity) may become significant when the fluid velocity is increased in the case of venous blood ejected from a working muscle. Since the flow is driven by a difference in total energy, blood can flow against gravity during muscle contraction. So, the calf muscle pump is the primary driving force to return blood from the deep veins of the lower leg (Miller et al., 2005). In agreement with this physical principle, some types of devices, based on the application of external pressure impulses to the calf muscles, have been designed to facilitate the venous return of blood to the heart in patients with paralyzed lower limbs (Malone et al., 1999; Manuello Bertetto et al., 2017).

Arterial pulsation during resting phases with normal breathing would be sufficient to maintain return flow from the lower limbs to the heart. However, much higher venous return occurs during muscle movements such as walking or foot flapping. Furthermore, a properly functioning calf pump is of vital importance to preserve the integrity of the microcirculation, by reducing distal capillary pressure when standing.

Considering a walking cycle, contraction of the calf muscles (muscle systole, corresponding to weight-bearing in Figure 3) compresses the intramuscular and deep veins, raising venous pressure and propelling blood in a central direction, while downward flow is prevented by the closure of distal valves. On subsequent muscle relaxation (muscle diastole, corresponding to elevation of the leg in Figure 3) the pressure gradient reverses and the proximal valve closes preventing retrograde flow, while the distal valve opens allowing venous refill from distal regions. In short, the legs veno-muscular pumps constitute a chain of actions due to their sequential activation during walking. So this anatomical district performs the role of a peripheral heart which greatly contributes to avoiding gravitational reflux during muscular relaxation (Uhl and Gillot, 2015).

The calf pump mechanism also involves superficial veins, connected to the deep system by perforating veins. The blood from the superficial venous system spills through to deep veins in a central direction. The pressure increase during muscle contraction is less pronounced within superficial veins: three times lower than in deep veins. Despite this pressure gradient, competent valves in perforating veins help to prevent reflux from the deep toward the superficial system. The venous pumping system during activity reduces both vein pressure and calf volume. On subsequent muscle relaxation, venous pressure falls below the pressure at rest. The fall is greatest in the deep veins, less in the superficial veins. In this phase perforating veins could allow flow from the superficial to the deep veins (Miller et al., 2005; Kotani et al., 2007; Keijsers et al., 2015; Joseph et al., 2016).

Immediately after exercise, the venous system is emptied by the calf pump function (more than 60% of venous blood is expelled in central direction) and the pressure within lower extremity veins is reduced from 100 mmHg to 15–30 mm Hg. This reduction is directly related to the height of the column of blood, which is divided in short segments by competent valves, assisting in relieving the vein wall from gravitational effects. However, with prolonged relaxation of the muscle pump, the veins slowly fill via arterial inflow and become distended, allowing opening of the valves and eventually pressure increase (Stranden and Kroese, 1998).

It has been found (Engelhorn et al., 2018) that the venous system of the lower limbs comprises the deep system, responsible for 85% of central venous drainage, and the superficial system, responsible for the remaining 15%, i.e., a very small percentage of blood is drained from the lower limbs superficial veins system toward the heart. Between these two systems there are several perforating veins that communicate, directly or indirectly, enabling flow to drain from superficial veins to deep veins. Strictly concerning the venous district of interest in our CFD modeling, i.e., that of the tibial veins, it has been found that perforators sit along the length of the anterior arch vein connecting it with the deep anterior tibial vein. Above the medial malleolus are the Cockett’s perforating veins connecting the posterior arch vein with the posterior tibial veins (Tretbar, 1995; Uhl and Gillot, 2015). So, taking into consideration that described above, it is possible that, concerning the walk simulation, in the valve chambers of our CFD model there might form a supplement of blood, in addition to that coming into the vein from the main input, which was drained from the perforating veins during the calf muscle relaxation phase. This hemodynamic peculiarity, not taken into consideration in our model, could make it less reliable, although *in vivo* blood flow measurements may suggest the existence of some compensatory effect due to outward flow in the perforating veins connecting the tibial veins with the saphenous one (Bjordal, 1972; Sarin et al., 1992*;* Tretbar, 1995; Recek, 2015) which, probably, could mitigate the effects of this lack in the computational model.

However, it must be said that the calf venous trunk is reduced to a cylinder with volume *V* = *πR*^2^*h* = 5.9 cm^3^ = 5.9 ml, being *R* = 0.25 cm and h = 30 cm. Therefore, the ejection fraction with a single muscle contraction in the model is equal to 0.2/5.9 = 3.3%. Assuming the volume *V* as representative of calf’s venous volume, the current model has the potential to replicate normal EF values (≥ 60% of total venous volume) by increasing either the aortic flow (by increasing the inlet radius) or the magnitude of displacement function for the calf muscle or, if necessary, combining both effects.

Unfortunately, all these refined venous mechanisms that allow the return of blood to the heart against gravity could be structurally and functionally damaged by several events of both exogenous and endogenous nature. In fact, individuals with amputated legs or spinal cord injuries, due to the lack of activity of the calf muscles (anatomical in the former case and functional in the second one and both often caused by an external accident), suffer from chronic diastolic insufficiency (Crisafulli et al., 2009) from which an inadequate cardiac output leading to a bad quality of life, can occur (Kurdibaylo, 1994; Huonker et al., 2003).

Even in lower limb fractures, the venous pump can become temporarily impaired due to the inability of calf muscles to contract, related to pain, swelling or cast immobilization (Massari et al., 2018). Moreover, up to 15% of the shaft fractures are complicated by compartmental syndrome which occurs when the pressure within the closed osteo-fascial muscle compartment of the calf rises above a critical level, and when the compartment pressure approaches diastolic pressure, both the capillary blood flow and venous flow cease (Schmidt, 2017). Another complication of lower limb fractures which can affect normal venous return, is the deep venous thrombosis which can eventually result in venous hypertension and chronic compartment syndrome (Engelbert and Turnipseed, 2014).

On the other hand, degenerative diseases of metabolic (Musil et al., 2011) or cardiovascular (Delis, 2005) origin can give rise to valvular dysfunction in each venous system (superficial, perforating or deep) and in combination (Williams et al., 2014).

Given this critical situation, the attention of researchers and clinicians who deal with hemodynamics is increasingly oriented toward the use of instruments and methods that provide numerically evaluable data. In response to this need, computational blood dynamics has been widely and successfully used for cardiac valves (Ferraresi et al., 1998, 1999) and concerning this matter the paper from Ariane et al. (2017) furnishes an extensive bibliography. On the contrary, there are far fewer papers concerning venous valves. In the majority of the venous valve simulations, the valves are fixed so that the complex interaction between the blood flow and the mobile valve flaps is lacking. Recently (Simao et al., 2016), flexible structures were investigated with the fluid structure interaction (FSI) method (Formaggia et al., 2007). Nevertheless, this kind of analyses could be restricted to few cycles or implemented with one symmetrical leaflet, mainly due to the intrinsic difficulty of the FSI method in simulating the contact between the two leaflets at the end of the closing phase. Van Loon (2010) used specific algorithms for the additional contact accounting for the mechanical contact between valve leaflets. Unfortunately, the implementation of these algorithms is complex and the frequency of the re-meshing close to the contact point remains the problem. In a very recent paper from Ariane et al. (2017) the hemodynamics in bicuspid flexible deep vein valves is modeled by means of discrete multi-physics together with an implemented agglomeration algorithm to account for blood accrual in the vein flow. These Authors demonstrated that both length and rigidity in the valve leaflets play a crucial role on mechanical stress and on blood flow stagnation. In fact, on the one hand the rigid and short membranes are inefficient in preventing blood reflux but, on the other hand, these conditions reduce the volume of stagnant blood so lowering the risks of thrombosis. Moreover, in venous valves the agglomeration of blood cells is mainly driven by stagnation rather than by mechanical stress. Authors concluded that a proprietary discrete multi physics model could predict, for that given valve, the location of maximum stagnation and give information which could be translated into a probability of thrombus formation for a specific individual. Unfortunately, with regards to this modelization approach the high degree of complexity greatly reduces its applicability in the clinical setting. Nevertheless, to simulate the interaction of valve structure with the surrounding flow, a computational model of a prosthetic valve based on realistic geometry and mechanical properties was developed by Tien et al. (2014). These simulation results were validated by experiments with a prosthetic bicuspid venous valve using the particle image velocimetry method (Saikrishnan et al., 2012) with high spatial and temporal resolution in a pulse duplicator. Limitation in these experiments arises by the validated FSI simulations which should enable future valve design optimizations that are needed for improved clinical outcome. An interesting research from Keijsers et al. (2015) concerned the assessment of the sensitivity of a venous valve model outputs in the presence of considerable input parameters. Using a 1D pulse wave propagation model of the tibial vein which included a venous valve, valve dynamics while head up tilt occurred were simulated. It was concluded that, for the output related to the opening state of the valve, the improved assessment of tibial vein radius and of the valve opening/closing pressure drop is most satisfying when simulating valve dynamics, since this resulted in a large reduction in the output uncertainty. However, in practice this could be achieved only by using ultrasound imaging of the veins and fluid structure interaction simulations to characterize detailed valve dynamics, respectively.

The above synthetic review of previous experimental results reasonably represents the current state of the art as regards the possibility of fluid dynamic modeling of the venous districts included in the valves’ boundary and, clearly, those results show that we are still far from being able to satisfactorily transfer the data of computational modelization to obtain unequivocal advantages in the clinical setting for venous valve incompetence and deep venous thrombosis.

Now considering our CFD model for tibial vein valves, its relevant originality consists in the fact that we first utilized a CFD simulation of the human tibial vein output by substituting the bicuspid valve model with spherical valve one. In this way we excluded from the computational model any imprecision due to the difficulties in simulating the complex interaction between the flow and the moving leaflets of a bicuspid bioprosthetic valve sample. In fact, it has been found (Ariane et al., 2017) that the rigidity and the length of the modelized valve leaflets operate in the opposite way to each other. Therefore, it would be ideal if both mechanical stress and stagnation in the flow were reduced in the same valve model. Indeed, the use of spherical valves in the circulatory system is not an absolute novelty (Dellsperger et al., 1983; Rashtian et al., 1986). In fact, a milestone paper published by Wogel and co-workers on the American Journal of Cardiology (Vogel et al., 1993) clearly highlighted the long lasting use of prosthetic spherical valves to substitute sick heart valves.

The fact that unidirectional spherical prosthetic valves are capable of operating at length without decreasing their fluid dynamic efficiency, appears from the data reported in a case study recently published (Yalcinkaya et al., 2016). In fact, these Authors reported that in 1973, a 10-year-old boy with a rheumatic mitral stenosis underwent this valve replacement with a Starr-Edwards caged-ball valve (Matthews, 1998). In June 2014 this patient was submitted to a trans-thoracic echocardiogram which showed a well-functioning mitral prosthetic caged-ball valve without regurgitation, a peak mitral velocity of 2.14 m/s, a moderate early diastolic gradient (18.37 mmHg), and a mild mean gradient (8.31 mmHg). Moreover, there was no paravalvular leak and the effective orifice area of the mitral valve was 2.5 cm^2^ or normal for this type of prosthesis. Cine fluoroscopic images of the mitral valve showed normal silicon-ball movements toward the cage in diastole and closure in systole. The patient was discharged from the hospital and had New York Heart Association class I functional capacity one month later.

Thanks to all the above cited experimental data we are confident that there can be no preconceived impediment in the use of spherical valves for a CFD model applied to the lower limb vein valves, as we have done in this paper.

Some investigators rely on criteria for abnormal duration and magnitude of retrograde venous flow to define pathological reflux due to valvular incompetence. However, the ejection fraction (EF), as the percentage of total venous volume (VV) ejected by the calf with each contraction, is a measure of the calf muscle pump efficiency and the principal parameter correlating with disease severity. Normal EF values are greater than 60%, whereas lower values can be correlated with patterns of chronic venous disease (CVD). Results of our CFD simulations seem to give some possible answers concerning the need for a more detailed knowledge regarding the effects of the incompetence of lower limb deep vein valves on the blood flow returning to the heart. In fact, in these CFD simulations it was found that venous flow in lower leg, at the level of the calf muscle, increases in response to the pumping action of the vessel wall during walking. Flow measurements performed with the simulation provide a net flow volume increased by a factor of 4 when compared to resting conditions (0.2 vs. 0.05 mL).

It is useful to quantify the deviation of flow volume rates provided by the present tibial vein CFD model from the actual values. Although the scarcity of literature on this hemodynamic theme, some experimental works are reported here below to address this issue.

Broderick et al. (2008), in four healthy subjects performing 10 toe curls at a pace of one contraction per second, assessed the total blood volume ejected from the posterior tibial veins by Doppler ultrasound measurements. A statistical analysis provided a median value of about 2.2 mL, corresponding to a blood flow of 0.22 mL per single contraction (i.e., 0.22 mL/s) which is very close to that obtained in simulation #3 (0.2 mL/s) for healthy subjects who takes a step lasting 1 s.

In the Broderick et al. experiments, the subjects contracted some toe muscles – such as the flexor digitorum brevis, quadratus plantae, abductor hallucis and the flexor digit mini brevis, instead of calf muscles – that should compress and empty the lateral and medial plantar veins, resulting likewise in the observed increase of posterior tibial vein output.

Furthermore, by means of duplex ultrasound technique, Sperber et al. (1996) measured both areas and blood flow velocities in posterior tibial veins of 10 anesthetized patients (before they underwent knee arthroscopy) having the mid-portion of the thigh supported in a loosely fitted leg-holder. The ultrasound data showed a considerable inter-individual variability regarding the two parameters considered. However, in one patient (patient # 9) the transverse tibial vein diameter was about 0.46 cm, namely very close to that in our CFD simulations (0.5 cm) – with a corresponding cross-sectional area of 0.16 cm^2^ – and a measured blood velocity of 1.6 cm/s. Both these variables are found to be not far from those provided by the CFD simulation of a healthy subject while taking a step (see simulation # 3). Remarkably, the resultant value of the tibial vein flow rate was Q = Av = 0.16 ⋅ 1.6 = 0.25 cm^3^/s = 0.25 mL/s, still in agreement with the proposed CFD model.

With the aim of determining impairment in lower limbs tissue perfusion in patients with peripheral arterial disease, Kalayci et al. (2015) measured mean flow rate together with blood flow velocity and diameter in the posterior tibial veins of 38 patients suffering from several artery diseases by means of the duplex ultrasonography. They found a mean flow rate of 15.21 mL/min, i.e., 0.25 mL/s that was not considerably different from the value of 0.20 mL/cycle in CFD simulations of healthy walking subjects. Moreover, these Authors also showed that with a mean tibial vein diameter of 2.44 mm (corresponding to an area of 4.67 mm^2^) the mean blood velocity in these veins was of 9.3 cm/s. An extrapolation is possible considering that the tibial vein diameter of tested subjects ranged from 1.3 to 5.4 mm. The related mean value of the vessel cross sectional area was found to be 19.62 mm^2^, which corresponds – considering as a constant the flow rate of 0.25 mL/s – to a blood velocity v = Q/A = 0.25 cm^3^/s/0.196 cm^2^ = 1.27 cm/s, quite close to CFD simulation results for walking subjects.

Finally, by using a novel real-time magnetic resonance imaging method for velocity-encoded phase-contrast, Joseph et al. (2016) conducted clinical studies 8 healthy subjects in supine position performing about 9 consecutive foot flexion and extension, each lasting about 1 s. These trials showed an integrated flow volume output of 1.6 mL in the right posterior tibial vein, which corresponded to 1.6 mL/9 cycles = 0.186 mL of blood volume ejected in 1 s (duration of one calf contraction/relaxation cycle), which is still in agreement with CFD model simulations.

Based on the findings of the experimental studies considered above, it can be reasonably believed that the CFD model, here used to simulate the venous return in lower limbs can be considered as reliable.

When valvular dysfunctions are studied by CFD simulations during walking, a reduced net flow in upward direction is observed in the case of an incompetent proximal valve with flow volume of 0.14 mL per cycle (against 0.2 mL for simulated physiological conditions), or a 30% reduction in the venous flow. This model result can be compared with the mean percentage EF reduction of about 25% in CVD limbs compared with healthy limbs (Araki et al., 1994).

This reduction is due to the strong bidirectionality of the flow, where the magnitude of diastolic downward flow becomes comparable to the systolic upward flow. This demonstrates that venous disease is characterized by the diastolic phase of the calf muscle (Stranden and Kroese, 1998). In fact, no significant changes in the flow volume are revealed in the case of incompetent distal valve by our CFD modeling. The present findings suggest that the valvular dysfunction of the calf pump is determined by the proximal valve, while the influence of the distal valve apparently is negligible.

This study essentially allows for increased understanding of the effects of venous valve incompetency on the returning blood to the heart. Nevertheless, one could use this model to better understand clinically pressing concerns such as deep vein thrombosis. In fact, often a valve’s incompetence also results in blood stagnation within the valve site, from which blood clotting could easily result, leading to a venous thrombosis.

The latter vascular dysfunction is particularly pertinent to an acute traumatic occurrence as in the tibial fracture. In fact, these limb fractures are quite frequent, i.e., about 1% of all fractures (Elsoe et al., 2015; Tan et al., 2018) and, especially in sports that mainly utilized legs such as soccer, it has been found that tibial fractures could reach 3.6% of all those occurring while playing (Schiffner et al., 2019). Restricting the observation field to lower limb bones fracture, tibial ones are among those that can give rise to serious problems in the blood flow of their veins, hence the remarkable risk of intra-vascular thrombus formation. In fact, it has been found that about 3% of patients who underwent tibial plateau fractures developed a venous thromboembolism as post-operative complications (Kugelman et al., 2017). Moreover, in a population of about 100 patients who have had the operative fixation of fractures of the lower extremity distal to the hip, about 28% of them developed a deep-vein thrombosis, 60% of which had tibial fractures (Abelseth et al., 1996).

However, our CFD model can also be applied in chronic degenerative diseases such as, for instance, those related to body fat accumulation. So, obesity is another pathological condition recognized as a strong and independent risk factor for deep venous thrombosis. In fact, many studies have reported a 2- to 3-fold increased risk of deep venous thrombosis in people with a BMI > 30 kg/m^2^ compared with normal-weight subjects (Stein et al., 2005; Borch et al., 2011). Due to excess abdominal fat, central obesity is believed to be associated with increased intra-abdominal pressure. Indeed, in obese patients it has been shown that the pressure in the ilio-femoral vein was significantly higher than in non-obese individuals (Arfvidsson et al., 2005). Furthermore, weight reduction by surgery decreases urinary bladder pressure, an indirect marker of intra-abdominal pressure (Sugerman et al., 1997). So, an elevated intra-abdominal pressure might be transmitted to the leg extremities (i.e., to the tibial veins) by the femoral veins, leading to venous stasis and distensions of the veins of the lower limbs, favoring in this way thrombosis and venous valve dysfunction. The femoral vein flow was studied by Willenberg et al. (2010) which found that the blood peak velocity was significantly higher in healthy (14.8 ± 7.2 cm/s) than in obese participants (10.8 ± 4.8 cm/s). Moreover, the femoral vein diameter was significantly smaller in the control (7.1 ± 1.6 mm) than in obese participants (8.5 ± 2.2 mm). This could be interpreted as a result of elevated, abdominal fat-depending, intra-abdominal pressure transmission to the femoral veins which leads to vein wall distension along the entire vascular tree downstream and, on the basis of the Bernoulli’s law, a reduced forward flow velocity might occur as a consequence of this altered venous regimen. As is known, a slowdown in blood flow can trigger the reaction cascade of coagulation justifying the elevated risk of deep vein thrombosis in obese patients.

In the light of the above described scenario, we are confident that the CFD model we used and tested here, although somewhat simplified and not providing the ideal conditions to simulate real fluid dynamic behavior of the tibial veins, can already represent a computational device that helps physicians engaged in clinical matters implementing leg vein diseases to improve their diagnostic and therapeutic efficacy. Consequently, on the basis of the numerical data reported here under simulation conditions of the trend of blood flow with competent or insufficient venous valves of the tibial veins, we can reasonably consider our CFD model to be an innovative and practical device for the study of leg deep veins.

In conclusion, we believe that this CFD model can be used in helping to predict the chances of survival against the risks of thrombosis and mortality in those patients that show leg deep veins with valves incompetence. Briefly, the results of this simulation, in association with other clinical data, highlights the possibility to have a support for the development of software assistance solutions for patients’ therapy by providing useful information regarding the hemodynamic behavior associated with the evolution of deep leg vein diseases.

## Study Limitations

As this work represents a modeling study of blood flow in peripheral veins, it has limitations due to the simplifications of the simulated venous network in the lower leg segment. Future improvements may consider the transformation of the calf trunk into a solid, elastic domain in which deformations would result from applied external pressures, and the possibility of simulating the perfect closure of the valves.

## Data Availability Statement

The datasets generated for this study are available on request to the corresponding author.

## Author Contributions

AlC conceived the original project. GN and AM performed the experiments and acquired the data. AnC and GM contributed to the design of work as well as the analysis and interpretation of the data especially with regard to orthopedic applications. FV proceeded to direct the results also toward overweight patient applications. AlC, AD, and AF supervised the study. GN and AlC wrote the manuscript. All authors contributed to the article and approved the submitted version.

## Conflict of Interest

AF was employed by the company Nomadyca Ltd., Kampala, Uganda. AC was employed by company: 2C Technologies Ltd. The remaining authors declare that the research was conducted in the absence of any commercial or financial relationships that could be construed as a potential conflict of interest.
